# Current and past smoking patterns in a Central European urban population: a cross-sectional study in a high-burden country

**DOI:** 10.1186/s12889-016-3216-5

**Published:** 2016-07-15

**Authors:** Narine K. Movsisyan, Ondrej Sochor, Eva Kralikova, Renata Cifkova, Hana Ross, Francisco Lopez-Jimenez

**Affiliations:** International Clinical Research Center, St. Anne’s University Hospital Brno, Pekarska 53, 656 91 Brno, Czech Republic; International Clinical Research Center, Department of Cardiovascular Diseases, St. Anne’s University Hospital Brno, Masaryk University, Brno, Czech Republic; Division of Cardiovascular Diseases, Mayo Clinic, Rochester, MN USA; Institute of Hygiene and Epidemiology, First Faculty of Medicine of Charles University and General University Hospital, Prague, Czech Republic; Centre for Tobacco Dependence of the 3rd Medical Department, First Faculty of Medicine of Charles University and General University Hospital, Prague, Czech Republic; Center for Cardiovascular Prevention of the First Faculty of Medicine, Charles University and Thomayer Hospital, Prague, Czech Republic

**Keywords:** Tobacco, Smoking cessation, Inequalities, Socioeconomic status, Cross-sectional survey, Central and Eastern Europe

## Abstract

**Background:**

Many studies have examined the socioeconomic variations in smoking and quitting rates across the European region; however, data from Central and East European countries, where the tobacco burden is especially high, are sparse. This study aimed to assess the patterns in current and past smoking prevalence based on cross-sectional data from a Central European urban population sample.

**Methods:**

Data from 2160 respondents aged 25–64 years in Brno, Czech Republic were collected in 2013–2014 using the Czech post-MONICA survey questionnaire to assess the prevalence of cardiovascular risk factors, including smoking status. The age- and sex-stratified randomized sample was drawn using health insurance registries. Descriptive statistics and quit ratios were calculated, and chi-square and multivariate logistic analyses conducted to examine relationships between current and past smoking and demographic (age, gender, marital status) and socioeconomic variables (education, income, occupation).

**Results:**

The prevalence of current and past smoking was 23.6 and 31.3 % among men and 20.5 and 23.2 % among women, respectively. Education reliably predicted smoking and quitting rates in both genders. Among men, being unemployed was associated with greater odds of smoking (OR 3.6; 1.6–8.1) and lower likelihood of quitting (OR 0.2: 0.1–0.6); the likelihood of quitting also increased with age (OR 1.8; 1.2–2.8). Among women, marital status (being married) decreased the odds of current smoking (OR 0.6; 0.4–0.9) and increased the odds of quitting (OR 2.2; 1.2–3.9). Quit ratios were the lowest in the youngest age group (25–34 years) where quitting was more strongly associated with middle income (OR 2.7; 95 % CI 1.2–5.9) than with higher education (OR 2.9; 95 % CI 0.9–8.2).

**Conclusions:**

Interventions to increase cessation rates and reduce smoking prevalence need to be gender-specific and carefully tailored to the needs of the disadvantaged groups of the population, especially the less well-off young adults. Future studies should examine the equity impact of the tobacco control policies and be inclusive of the Central and East European countries.

## Background

The number of premature deaths from coronary heart disease (CHD), for which tobacco use is a major risk factor, varies dramatically across Europe. There is a striking ten-fold difference in the CHD mortality rates between East and West European countries, and a seven-fold difference across Central and East Europe (CEE), being the highest in Belarus and lowest in Slovenia [1]. These deaths can be prevented by improving the quality of and access to healthcare, on one hand, and by implementing effective public health interventions to promote healthy behaviours and reduce the risk factors such as smoking, on the other [2]. Across Europe, the smoking prevalence declined from 2000 to 2010 but at different rates [3]. Furthermore, some studies have suggested that, because of lower smoking cessation rates among disadvantaged populations, the existing differences in smoking rates across socioeconomic strata may have increased [4, 5].

Countries in Central and East Europe have higher smoking rates but also lower quitting rates [4] and the age, gender, and educational patterns of smoking prevalence there differ from those in the west and north. They also had largest inequalities in smoking-related mortality, but smoking inequalities were relatively small, with the exception of the Czech Republic where larger differences in male smoking rates across socioeconomic strata were found [2]. The Czech Republic has remarkably high cardiovascular mortality rates (twice the EU average) despite the sharp decline following the post-communist transformation [3, 6]. The reduction in the prevalence of cardiovascular risk factors, such as hypertension, dyslipidemia and smoking, from 1985 to 2007 along with the advances in medical care, played a major role in this decline [7–9]. Though the smoking prevalence fell from 45.0 to 30.5 % among men, female smoking remained relatively stable (overall mean prevalence 23.9 %) [8]. A national survey in 2013 found rates of 36.4 and 23.7 % for current smoking in men and women (aged 15+), respectively [10]. According to EuroStat (2012), the Czech Republic is one of the few EU countries with rising smoking rates. Therefore, there is an untapped potential to improve cardiovascular and other chronic disease mortality through interventions aimed at smoking prevention and cessation in the population. It is important, however, that these interventions account for differences in the smoking and cessation rates among the whole socioeconomic spectrum and strive to reduce instead of widening them unintentionally.

The city of Brno, the second largest city in the Czech Republic, with its high proportion of university graduates, has a significant growth potential [11]. However, the rapid urban development may lead to re-emerging of socioeconomic and health disparities. The purpose of the present study was to identify the socioeconomic and demographic factors associated with the current and past smoking rates in the Brno population and propose recommendations for effective and targeted population-based interventions to reduce smoking prevalence in the population. This study is part of the larger cross-sectional survey Kardiovize Brno 2030 to examine the prevalence of cardiovascular risk factors in the Brno population [12].

## Methods

### Study population

The Kardiovize cross-sectional survey was conducted in 2013–2014 in Brno, Czech Republic to assess the prevalence of cardiovascular risk factors in the Brno adult population aged 25–64 years. As of January 1, 2013, Brno had a population of 373,327 [11].

### Study sampling and recruitment

The survey sampling was done in January 2013 with the technical assistance from the largest health insurance (state-run) company in the Czech Republic, and using the registries of other health insurance companies. One of the companies declined to cooperate, thus about 8.9 % of the Brno population did not have a chance to participate in the study. Registration with a health insurance company is mandatory in the Czech Republic [11]. No a priori calculation of the sample size was done. The study set a target of selecting about 1 % of the adult population (25–64 years) as in the WHO MONICA study [8]. A random sample of 3,600 Brno permanent residents stratified by gender and age group was drawn from the registries of the health insurance companies to adjust for the expected 64.4 % response rate based on the Czech MONICA and Czech post-MONICA studies. The health insurance companies created a stratified sample, maintained the list of study subjects, and mailed invitation letters with a detailed description of the study goal and health screenings developed by the research team to the potential participants to NT13434-4/2012ensure the confidentiality of personal information. If interested, the invitees were able to provide their preliminary consent through one of the following options: phone call, e-mail, or prepaid mail. The invitation letters were mailed in January 2013, with two reminder mailings following in the case of non-response. Following the same procedure, another random sample of 3,077 was drawn in April 2014 to reach the target of 1 % of the city adult population that was met on December 19, 2014. Based on the two sample sets with a total of 5,090 randomly selected persons, the estimated overall response rate was 33.9 %. Despite the rather low response rate, the final sample size of 2,160 was large enough to ensure the representativeness of various sociodemographic strata in the sample.

### Measurements

The study included a face-to-face health interview using an extended version of the Czech post-MONICA study questionnaire, physical examination, laboratory testing as well as other health exams [8]. We also validated smoking status by objective measurement of carbon monoxide (CO) levels in expired breath using a Bedfont Micro+ Smokelyzer (Bedfont Scientific Ltd, Kent, UK) [13]. Trained nursing and medical personnel carried out the measurements and exams using a standard study protocol.

Smoking status was categorized as never smoking, current smoking and past smoking based on two questions: “Have you smoked more than 100 cigarettes in your life?” and “I used to smoke till the age of …”. Current smoking was defined as smoking either daily or less than daily (occasionally) up to his/her current age and having smoked more than 100 cigarettes in a lifetime. Past smoking was defined as having smoked more than 100 cigarettes and stopped smoking for at least 1 year. Quit ratio was calculated as the ratio of the number of past smokers over the number of ever-smokers, i.e. total of current and past smokers [4].

The respondents’ socioeconomic status (SES) was assessed by three main indicators, i.e., educational attainment, occupational status, and monthly household income. The participants were asked if they had one of the six levels of education that were further grouped into three categories: primary (complete/incomplete primary and apprenticeship without a school-leaving exam), secondary (apprenticeship with a school-leaving exam, secondary, and specialized secondary), and higher education. Occupational status was assessed based on the reported occupation and coded as manager/self-employed, skilled/professional, non-skilled/junior, and unemployed. The respondents were asked about their net monthly household income in local currency (Czech crown/CZK) by selecting one of the five categories (less than 15,000; 30,000; 45,000; 60,000, and more) that were further grouped into three categories: low (<30,000), middle (30,000–45,000), and high (45,000+).

Other SES variables included employment/allowance type (full-time, part-time, disability pension, retirement pension, household keeping), and household size (small/1–2 persons, medium/3–4 persons, large/5–6 persons). Marital status included single, married, divorced/widowed categories. The participant’s age was recorded as a continuous variable and further categorized into one of the four age groups (25–34 years, 35–44 years, 45–54 years, and 55–64 years).

### Data collection and statistical analysis

The face-to-face interviews and examinations took place in a hospital setting by the research staff who entered the collected data using the RedCap (Research Electronic Data Capture) web-based application [14]. The data were cleaned after the completion of the survey in December 2014 using a standard protocol. Data analysis was performed with STATA statistical software (StataCorp 2011, Release IC 11, College Station, Texas, USA) and included descriptive statistics, chi-square tests, and simple and multivariable logistic regression analyses. The logistic regression was fitted with smoking status as a dichotomized dependent variable (current smoking and the other two categories, i.e. never smoking and past smoking), and categorical (education, income) or dichotomized (occupation, marital status, household size) independent variables, except for the variable of age (continuous).

## Results

The survey was completed by a total of 2160 participants aged 25–64 years with a mean age of 47.3 years (±11.3), of which 54.8 % were women. The majority were full-time employed (75.6 %), married (62.1 %), and had never smoked (51.5 %). About 43 % had a net household income less than 30,000 CZK (≈1,095 EUR) and 50.8 % lived in a family of 1–2 persons (Table 1). The proportions of men and women in the study did not differ across the age groups (*p* = 0.25).

**Table 1:** Participants’ sociodemographic characteristics by gender

Variable, *N* (%)	Total *n* = 2160	Men *n* = 977	Women *n* = 1183	P*
Age, years				0.25
25–34	347 (16.1)	168 (17.2)	179 (15.1)	
35–44	357 (24.7)	252 (25.8)	285 (24.1)	
45–54	568 (26.3)	256 (26.2)	312 (26.4)	
55–64	708 (32.8)	301 (30.8)	407 (34.4)	
Marital status				<0.001
Single	408 (19.0)	207 (21.3)	210 (17.0)	
Married	1336 (62.1)	639 (65.7)	697 (59.1)	
Divorced	341 (15.8)	116 (11.9)	225 (19.1)	
Widowed	68 (3.2)	11 (1.1)	57 (4.8)	
Education				<0.001
Primary	427 (19.8)	203 (20.8)	224 (19.0)	
Secondary	889 (41.3)	350 (35.9)	539 (60.6)	
Higher	836 (38.9)	422 (43.3)	414 (35.2)	
Household income, CZK				<0.001
Low (<30 000)	877 (43.4)	315 (34.1)	562 (51.2)	
Middle (30 000–45 000)	632 (31.3)	313 (33.8)	319 (29.1)	
High (45 000+)	513 (25.4)	297 (32.1)	216 (19.7)	
Occupation				<0.001
Manager/self-employed	692 (32.3)	417 (42.9)	275 (23.5)	
Skilled/professional	1234 (57.6)	479 (49.2)	755 (64.5)	
Non-skilled/junior	141 (4.9)	47 (4.8)	67 (8.0)	
Unemployed	77 (3.6)	30 (3.1)	47 (4.0)	
Employment type				<0.001
Full-time	1559 (75.6)	813 (86.5)	746 (66.5)	
Part-time/Disability	172 (8.34)	54 (5.7)	118 (10.5)	
Retired/Housekeeping	331 (16.1)	73 (7.8)	258 (23.0)	
Household size, persons				0.04
Small (1–2)	1094 (50.8)	467 (47.9)	627 (53.3)	
Medium (3–4)	950 (44.1)	457 (46.8)	493 (41.9)	
Large (5–6)	108 (5.0)	52 (5.3)	56 (4.8)	

P*: *p*-value, Pearson chi-square test

Of the 2160 records, six with missing data on smoking status and socioeconomic variables, were excluded from the analysis. One subject with missing data on smoking status was included in the analysis based on the verified expired CO level. The results of the expired CO measurement were available for 2,149 subjects. As expected, the CO levels differed substantially by self-reported smoking status and number of cigarettes smoked. Based on the cutoff of 6 ppm, the number of persons with inconsistent self-reported and CO data comprised 5.7 % (122) of the total sample, 7.0 % (68) for men and 4.6 % (54) for women.

Self-reported current smoking prevalence was 21.9 % in the study population sample (23.6 % male, 20.5 % female) and past smoking was 27.0 % (31.3 % male, and 23.2 % female) The self-reported smoking status differed significantly between men and women in the study, never smoking being reported by 56.1 and 45.1 % of female and male participants, respectively (*p* <0.001) (Table 2). All socioeconomic variables, except for employment type, were associated with smoking status in the chi-square analysis. The prevalence of current smoking was inversely proportional to household monthly income and educational attainment (p < 0.001 for trend for both). Smoking status differed in the chi-square analysis across age groups, marital status, occupation, and household size strata (Table 2). The rates of current smoking in men were higher among the unemployed (53.3 %) and those with primary education (39.4 %). Female smokers who had primary education or were divorced had the highest smoking rates of 29.0 % and 28.7 %, respectively (Table 3).

**Table 2:** Participants’ sociodemographic characteristics by smoking status

Variable, N (%)	Total number	Never smoking	Current smoking	Past smoking	P*	Quit ratio
Gender					<0.001	
Male	976	440 (45.2)	230 (23.5)	306 (31.3)		57.1
Female	1178	661 (56.3)	242 (20.5)	275 (23.2)		53.2
Age, years					<0.001	
25–34	347	188 (54.2)	78 (22.5)	81 (23.3)		50.9
35–44	536	312 (58.2)	105 (19.6)	119 (22.2)		53.1
45–54	567	290 (51.2)	127 (22.4)	150 (26.4)		54.2
55–64	704	311 (44.2)	162 (23.0)	231 (32.8)		58.8
Marital status					<0.001	
Single	408	223 (54.7)	103 (25.2)	82 (20.1)		44.3
Married	1333	695 (52.1)	254 (19.1)	384 (28.8)		60.2
Divorced	339	142 (41.9)	97 (28.6)	100 (29.5)		50.8
Widowed	67	36 (53.7)	18 (26.9)	13 (19.4)		41.9
Education					<0.001	
Primary	427	145 (34.0)	145 (34.0)	137 (32.0)		48.6
Secondary	889	418 (47.0)	215 (24.2)	256 (28.8)		54.4
Higher	836	536 (64.1)	112 (13.4)	188 (22.5)		62.7
Household income, CZK					0.001	
Low (<30 000)	877	415 (47.3)	224 (25.5)	238 (27.2)		51.5
Middle (30 000–45 000)	632	319 (50.5)	128 (20.2)	185 (29.3)		59.1
High (45 000+)	513	293 (57.1)	91 (17.7)	129 (25.2)		58.6
Occupation					0.036	
Manager/self-employed	692	348 (50.3)	139 (20.1)	205 (29.6)		59.6
Skilled/professional	1234	641 (51.9)	274 (22.2)	319 (25.9)		53.8
Non-skilled/junior	141	75 (53.2)	29 (20.6)	37 (26.2)		56.1
Unemployed	77	31 (40.2)	28 (36.4)	18 (23.4)		39.1
Employment type					0.317	
Full-time	1559	802 (51.4)	343 (22.0)	414 (26.6)		54.7
Part time or disability	172	92 (53.5)	38 (22.1)	42 (24.4)		52.5
Retired	331	168 (50.8)	60 (18.1)	103 (31.1)		63.2
Household size, persons					0.001	
Small (1–2)	1094	514 (47.0)	268 (24.5)	312 (28.5)		53.8
Medium (3–4)	950	519 (54.6)	185 (10.5)	246 (25.9)		57.1
Large (5–6)	108	67 (62.0)	18 (16.7)	23 (21.3)		56.1

P*: *p*-value, Pearson chi-square test

**Table 3:** Quit ratios by selected sociodemographic characteristics and gender

	Men (*n* = 976)				Women (*n* = 1178)			
Variable, *N* (%)	Current smoking (*n* = 230)	Past smoking (*n* = 306)	Quit ratio	P*	Current smoking (*n* = 242)	Past smoking (*n* = 275)	Quit ratio	P*
Age, years				0.03				0.9
25–34	43 (25.6)	39 (23.2)	47.6		35 (19.6)	42 (23.5)	54.6	
35–44	56 (22.2)	59 (23.4)	51.3		49 (17.3)	60 (21.1)	55.1	
45–54	61 (23.8)	81 (31.6)	57.3		66 (21.22)	69 (22.2)	51.1	
55–64	70 (23.3)	127 (42.3)	64.5		92 (22.8)	104 (25.7)	53.1	
Marital status				0.04				0.01
Single	55 (26.6)	45 (21.7)	45.0		48 (23.9)	37 (18.4)	43.5	
Married	138 (21.6)	214 (33.5)	60.8		116 (16.7)	170 (24.5)	59.4	
Divorced	33 (28.45)	41 (35.3)	55.4		64 (28.7)	59 (26.5)	48.0	
Widowed	4 (36.4)	4 (36.4)	50.0		14 (25.0)	9 (16.1)	39.1	
Education				0.06				0.01
Primary	80 (39.4)	78 (38.4)	49.4		65 (29.0)	59 (26.3)	47.6	
Secondary	87 (24.9)	127 (36.3)	59.4		128 (23.8)	129 (23.9)	50.2	
Higher	63 (14.9)	101 (23.9)	61.6		49 (11.8)	87 (21.0)	64.0	
Household income, CZK				0.5				0.1
Low (<30 000)	89 (28.3)	107 (34.0)	54.6		135 (24.0)	131 (23.3)	49.3	
Middle (30 000–45 000)	70 (22.4)	107 (34.2)	60.5		58 (18.2)	78 (24.5)	57.4	
High (45 000+)	57 (19.2)	78 (26.3)	57.8		34 (15.7)	51 (23.6)	60.0	
Occupation				0.002				0.1
Manager/self-employed	84 (20.1)	142 (34.1)	62.8		55 (20.0)	63 (22.9)	53.4	
Skilled/professional	117 (24.4)	145 (30.3)	55.3		157 (20.8)	174 (23.1)	52.6	
Non-skilled/junior	12 (25.5)	15 (31.9)	55.6		17 (18.1)	22 (23.4)	56.4	
Unemployed	16 (53.3)	4 (12.2)	20.0		12 (25.5)	14 (29.8)	53.9	

P*: *p*-value, Pearson chi-square test

The associations between current smoking and SES variables including education, household income, occupation, and household size remained significant in the simple logistic analysis. In the multivariable logistic model using education, income, occupation, and household size as independent variables and adjusted for gender, age and marital status, current smoking was inversely associated with higher (than primary) educational attainment, and associated with being unemployed. Though covariates such as education, income, and occupation may correlate with each other, the correlation coefficients in our data were low except for income and education (*r* = 0.36). The diagnostic test did not reveal problems with collinearity in the model.

We also fitted the final multivariable logistic model separately for men and women. In the gender-stratified analysis, an association between smoking and educational attainment was found for both genders; with the magnitude slightly higher for secondary education in men (Table 4). The occupation (unemployment) showed no relationship with smoking among females. On the contrary, the odds of current smoking were remarkably high (OR = 3.6; 95 % CI 1.6–8.1) among unemployed men. Being married had a protective effect for current smoking among females (OR = 0.6; 95 % CI 0.4–0.9) (Table 4).

**Table 4:** Adjusted ORs and 95 % CIs for current and past smoking by gender (multivariable logistic analysis)

Variable	Current smoking vs. never and past smoking		Past smoking vs. current smoking	
	Adjusted^a^ OR (95 % CI)		Adjusted^a^ OR (95 % CI)	
	Men	Women	Men	Women
Age, years				
<55	1	1	1	1
55–64	0.8 (0.5–1.2)	1.0 (0.7–1.4)	1.8 (1.2–2.8)	1.0 (0.6–1.5)
Marital status				
Single	1	1	1	1
Married	1.0 (0.6–1.6)	0.6 (0.4–0.9)	1.5 (0.9–2.6)	2.2 (1.2–3.9)
Divorced/widowed	1.4 (0.8–2.5)	1.0 (0.6–1.6)	1.3 (0.7–2.4)	1.4 (0.8–2.6)
Education				
Primary	1	1	1	1
Secondary	0.5 (0.3–0.7)	0.7 (0.5–0.9)	1.6 (1.0–2.5)	1.2 (0.7–1.9)
Higher	0.3 (0.2–0.4)	0.3 (0.2–0.5)	1.8 (1.1–3.0)	2.1 (1.2–3.7)
Occupation				
Employed	1	1	1	1
Unemployed	3.6 (1.6–8.1)	1.2 (0.6–2.6)	0.2 (0.1–0.6)	1.2 (0.5–2.6)

^a^adjusted for age, marital status, education, occupation, household income, and household size

Similarly, we ran a multivariable logistic regression to identify the socioeconomic variables associated with past smoking (quitting) (Table 4). Past smoking was directly associated with secondary (OR = 1.6; 95 % CI 1.0–2.5) and higher (OR = 1.8; 95 % CI 1.1–3.0) education among men, but exclusively with higher education (OR = 2.1; 95 % CI 1.2–3.7) among women. Older men and married women who had smoked in their lifetime had significantly higher odds of being former smokers at the time of the survey (OR = 1.8; 95 % CI 1.2–2.8) and (OR = 2.2; 95 % CI 1.2–3.9), respectively (Table 4). In the logistic regression stratified by age groups and adjusted for education, household income and size, quitting in the youngest adults (25–34 years) was more strongly associated with middle income (OR = 2.7; 95 % CI 1.2–5.9) than with higher education (OR = 2.9; 95 % CI 0.9–8.2) (data not shown).

The quit ratios differed by gender, age and socioeconomic variables (Tables 2 and 3). Younger men had lower quit ratios compared to older ones (47.6 vs. 64.5; *p* <0.05) while no differences in quit ratios were found across age groups for female smokers.

## Discussion

This study found a prevalence of self-reported current smoking of 21.9 % (23.6 % male, 20.5 % female) while the past smoking prevalence was 27.0 % (31.3 % male, 23.2 % female). Of the three common dimensions of SES, i.e. income, education, and occupation, only education showed a strong and stable inverse association with current smoking. Occupation was associated with male smoking, with unemployed men having more than triple odds of current smoking. The determinants of past smoking were similar to those of current smoking, except for age that appeared to increase the likelihood of quitting among men. Marital status was a significant predictor of current and past smoking among women, with higher odds of smoking and lower odds of quitting for singles. The likelihood of quitting for men and women increased stepwise with the level of education; for women, it was the higher level education that mattered while, for men, the odds of quitting started to increase with secondary education. Thus, the determinants of smoking and past smoking in this study were gender-specific. Being married predicted current and past smoking for women but not for men. Unemployment was linked to current and past smoking among men, but not women. Older age was predictive of quitting among men. These gender-related differences in determinants of smoking and past smoking prevalence can possibly be explained by different social and family roles of men and women in the study population.

The quitting patterns across Europe have been less studied than those of smoking. The studies generally found greater odds of cessation among higher educated smokers [4, 15] whereas smoking was shown to be inversely related to labour market attachment and earnings [16]. Some suggested that the inequalities in quitting rates widened over time, arguably as a result of implemented tobacco control policies [5, 17, 18].

Little is known about the smoking cessation prevalence in Central and Eastern Europe. The Czech Republic is one of these countries with highest cardiovascular mortality in the region, determined, to a large extent, by behavioural risk factors, including smoking [19]. To our knowledge, this is one of a few studies in Central and East European populations and the first from the Czech Republic that examined the past smoking prevalence and its socioeconomic determinants, using cross-sectional data, in the peer-reviewed literature. The social patterns in smoking rates in this study were broadly similar to findings from other high-income European countries where smoking was strongly and inversely associated with education in both genders, with the exception of southern Europe where this association was either weak or reversed in women [20]. Studies carried out in the Central and East European countries showed inconsistent associations of current and past smoking with gender, age, and marital status. This could be explained by different smoking/quitting patterns across the region corresponding to a different pace of transition between the stages of smoking epidemics, and also by methodological heterogeneity [21–26].

The quit ratio of 55.2 (57.1 male and 53.2 female) in our study was remarkably higher than those in former Soviet Union (fSU) and Baltic countries and more consistent with estimates from two comparative European studies that were inclusive of East European countries [4, 27]. Our estimates were closer to quit ratios in southern Europe and yet lower than in countries in the north, as in the study by Shaap et al. In the latter, the quit ratios in the Czech sample (2002) were slightly lower than our estimates while the magnitude of association between education and quitting was consistent with our findings [4]. Though it could be contemplated that the quit ratio in the Czech population increased over the last decade while its social gradient remained stable, this conclusion should be treated with caution because the estimates could have been affected by the sample size/representativeness or other issues, such as inclusion/exclusion of occasional smokers.

Our study defined past smoking as having stopped smoking for at least 1 year; however, people may stop smoking for shorter terms. Regardless of the socioeconomic position, smokers might be equally likely to attempt to quit whereas the success of those quitting attempts is determined by many factors, including psychosocial (personality traits, self-efficacy, locus of control), economic (labour market attachment and earnings), environment (smoke-free policies), as well as health (tobacco addiction) and education [28, 29]. Education is believed to enhance the access to social, psychological and economic resources [30]. Not only those with higher education are more aware of health hazards of smoking, but they may have a greater control over their lifestyle choices.

This study found that the determinants of quitting differed for young adults. Thus, among smokers aged 25–34 years, quitting was more strongly associated with household income than with educational attainment. This can possibly be explained by a time lag between completion of the higher education and the ensuing social and economic rewards.

### Limitations and strengths

The study used cross-sectional data to assess the prevalence of current and past smoking and their socioeconomic correlates whereas the incidence rates are equally important for understanding the patterns of the smoking epidemic. The study was carried out in a single urban setting, the city of Brno, admittedly representing the urban population in the Czech Republic (except for Prague, the countryʼs capital, with a population over one million, other ten major cities have populations of only 100–300 thousand) [31].

The low response rate could have affected the study representativeness resulting from self-selection bias. The response rate of 33.9 % was markedly lower than was projected based on the Czech post-MONICA study [8]. The following differences between two studies could have contributed to the low response: a) using a hospital setting for the health interviews and measurements, and b) not offering incentives to participants of the Kardiovize project. Low response rates in the Czech population (ranging from 30.6 to 55.0 %) were also reported by other contemporary studies [32, 33]. The study sample, however, was large enough to ensure the gender (54.8 % female as compared to 51.7 % in the Brno population) and middle age group representativeness; while the youngest (24–35 years) and the oldest (55–64 years) were, respectively, under- and over-represented. This could have limited the generalisability of the study findings and affected our estimates of current smoking (23.6 % male and 20.5 % female) that were notably lower than those found in the 2013 national survey (36.4 % male and 23.7 % female) [10]; however, the age limits differed in these two studies. We were not able to obtain the data on non-participants because the initial contact was made by the health insurance company. However, other studies found that the participants in epidemiologic studies were more educated and had healthier behaviours than the general population [34]. Therefore, our findings could have underestimated the smoking prevalence while overestimating the quit ratios; however, the self-selection bias should have not greatly affected the associations with socioeconomic variables while some underestimation of the magnitude of these associations is still possible.

Other methodological issues may include measurement and misclassification bias. Thus, occupation was a significant contributor when measured in the divergent categories, i.e. employed vs. unemployed, but not across the hierarchical groups, such as managers, skilled, and non-skilled workers. In this respect, a measurement error in assessing the occupational status (self-report, use of non-standard question) cannot be totally excluded. The negative findings related to the material determinants could have been affected due to misreporting, including differential misreporting. However, the non-response rate to the income question was only 6.4 % in the sample and it is unlikely that this alone would explain the results. We used the monthly household income adjusted for the consumption unit, i.e., household size, as a proxy measure of material wellbeing. However, using register-based income data could minimize the non-response and reporting biases while the combination with other proxy measures such as ownership of assets/goods (home, car, etc.) could provide a more comprehensive measure of material well-being [35]. Remarkably, material indicators have been consistently shown to be less important causes of health and smoking inequalities in the Czech Republic [7].

This study used a compositional approach in assessing the inequalities [36] and did not account for contextual, such neighbourhood/area [33, 37] or psychosocial factors, such as level of control/autonomy, that may have also contributed to the variations in current and past smoking rates [30].

This research comes from the Kardiovize Brno 2030 cross-sectional sample of 2,160 Brno residents randomly selected after stratification by age and gender from a comprehensive list of health insurance companies. The study provides a snapshot of the patterns of current and past smoking in an urban population sample from the Czech Republic in the decade since joining the EU community. It is the first population-based study to examine the quit ratios in the Czech Republic and the association of past smoking with socioeconomic status.

This study used a slightly modified Czech post-MONICA questionnaire whose validity and reliability had been tested in earlier studies [8]. Unlike many others, this study used more than one socioeconomic indicator to assess the relationships of socioeconomic status with smoking and quitting rates [35]. To our knowledge, this is the first population-based survey in Central and Eastern Europe that used biochemical validation of smoking status in the study sample.

### Implications

The impact of tobacco control policies in reducing smoking in many countries where these policies have been implemented is rather convincing; however, the positive equity impact has not been consistently demonstrated [38]. Equity-oriented policy approaches include targeted, i.e. reaching out disadvantaged smokers, and universal strategies, such as an increase in tobacco product prices/taxation [39]. The latter was shown to reduce smoking at higher rates among the less advantaged social groups and was also effective in preventing youth smoking [40, 41]. A unique case of the universal strategy with a potential for targeted interventions delivery is primary care [42]. Unlike many other East European countries, the Czech Republic timely introduced universal health coverage, along with other anti-poverty policies, and made a smoother societal transition [43, 44]. The Czech primary health care system has already a few preventive programs in place. However, these programs need to be re-oriented toward evidence-based preventive services, including targeted outreach to the least advantaged groups such as those with lower education, unemployed, or living alone.

## Conclusions

Education was the most stable and salient predictor of current and past smoking while the other correlates differed by gender in the study population. Our findings suggested that the differences in smoking/quitting rates start to accumulate already in early adulthood. Reducing smoking among less advantaged young adults should become a priority and be addressed by policy measures, such as tobacco taxation. Gender-specific interventions might be warranted to increase quitting in middle-age smokers. It is also important that future research will evaluate equity-oriented outcomes of such interventions and policies. Finally, further research using a standardized approach is needed to better understand the divergent trends in current and past smoking in Central and Eastern Europe.

## Abbreviations

CEE, Central and Eastern Europe; CHD, coronary heart disease; CO, carbon monoxide; CZK, Czech crown; fSU, former Soviet Union; MONICA, MONIitoring trends and determinants in CArdiovascular disease; SES, socioeconomic status; WHO, World Health Organization
